# Factors associated with successful treatment outcomes among tuberculosis patients in a district municipality of Vhembe, Limpopo

**DOI:** 10.4102/safp.v67i1.6030

**Published:** 2025-01-31

**Authors:** Busisiwe Mkhavele, Slindile Zondi, Lindiwe Cele, Mabina Mogale, Margeret Mbelle

**Affiliations:** 1 Department of Public Health, Faculty of Health Sciences, Sefako Makgatho Health Sciences University, Pretoria, South Africa

**Keywords:** tuberculosis, Vhembe district, tuberculosis cure rate, treatment success, treatment outcome

## Abstract

**Background:**

Treatment success rate is a critical indicator for monitoring the progress of tuberculosis (TB) treatment programmes at both the patient and population levels. It informs decisions about resource allocation and the effectiveness of TB control strategies. This study aimed to determine the level of TB cure rates and the factors associated with treatment success among TB patients receiving TB care in the Collins Chabane municipality, Limpopo province, South Africa.

**Methods:**

Medical records from April 2020 to March 2021 of 289 patients attending primary health care facilities, initiated on anti-TB treatment were reviewed. Descriptive statistics were used to analyse data and modified Poisson regression was used to determine factors associated with treatment success. Statistical software Epi Info was used for analysis.

**Results:**

Of the 289 TB cases, 282 (97.6%) were newly initiated on TB treatment. Of these, 37.0% were cured, followed by 29.0% who defaulted treatment, 22.3% who completed the treatment and 11.7% who died during treatment. The likelihood of successful treatment outcomes was significantly associated with marital status, supervised treatment and distance travelled to a health facility. A higher likelihood of success was observed among patients who were single and 27.0% of those had supervised treatments.

**Conclusion:**

Tuberculosis patients continue to die in the course of treatment. Supervised treatment is a predictor of successful treatment outcomes.

**Contributions:**

This study highlight the need for heightened advocacy for supervised TB treatment and increased effort to combat the death of patients while on TB treatment.

## Introduction

Routine assessment of treatment outcomes among patients who are on anti-tuberculosis (TB) treatment is useful for monitoring treatment progress among the patients who have TB. Tuberculosis treatment success rate (TSR) is a global indicator which is also used to track country progress towards meeting the targets and milestones as set out by the World Health Organization (WHO) and strategic partners. These targets are aimed at ensuring significant reductions in the number of TB infections and deaths, globally.^1,2,3^ The Stop TB partnership and the WHO have recommended achievement of 90% TSR by 2030; however, recent statistics indicate a global TSR of 87% in 2021, with countries in the African Region of the WHO reported to contribute most to the shortfall.^4^

Tuberculosis continues to be a public health burden in South Africa. The disease was listed among the top 10 leading causes of death in 2018, accounting for 44% of all deaths in the same year. The drivers of the TB epidemic in the country include among others, poor living conditions, late presentation to the health facility and high prevalence of human immunodeficiency viruses (HIV) infections. Human immunodeficiency virus was responsible for 55% of TB incidence and 66% of deaths among women in 2019 compared to 46% of TB incidence and 57% of deaths in males.^5,6,7^ The country fell short of the 90% TSR in 2021, achieving 79% TSR. This is despite South Africa’s concerted effort to combat the TB epidemic by designating TB control as one of its national priority programme (NPP). The country, in 2011, has also adopted the WHO endorsed GeneXpert (Xpert) machine for the initial testing of TB, to replace the sputum smear microscopy.^4,8,9^

Studies previously conducted in certain parts of South Africa have reported low TB cure rates of 65.8% in the Eastern Cape, 57.4% in KwaZulu-Natal and 57.9% in Limpopo.^10,11,12^ This is a disturbing finding, considering how South Africa is grappling with the dual burden of TB and the human immunodeficiency viruses (HIV) infections, with a national HIV prevalence of 13.7% in 2020, a small drop from a national estimate of 14.0% in 2017 and TB/HIV co-infection rate of 59.0% in 2019 among notified TB cases.^6,13,14^

Several studies that have investigated factors associated with TB treatment success have reported the age of patients, their place of residence, the distance from the health facility and gender as factors contributing to treatment success. Others have also reported influences of HIV co-infection and previous TB treatment with successful treatment outcomes.^15,16^ This study sought to determine the level of TB cure rates and the factors associated with treatment success among TB patients receiving TB care in Collins Chabane municipality.

## Research methods and design

### Study design

We used a descriptive cross-sectional study design and conducted a retrospective analysis of secondary data of TB patients who were initiated on TB treatment at primary healthcare facilities located in the Collins Chabane municipality, Vhembe district, Limpopo province.

### Study setting and study population

Collins Chabane municipality is one of four municipalities in the Vhembe district, located in the far north of Limpopo province, South Africa. This municipality is situated between greater Giyani, Thulamela and Makhado municipalities. It has an estimated population of 369 876 people, with Africans constituting the highest number of people. The majority of the residents live in traditional, brick-built homes. There are 33 primary health care (PHC) facilities and 1 hospital in the area. The PHC facilities provide TB treatment as well as other services. Disease profiling of the municipality area reveals that pulmonary tuberculosis (PTB), diabetes mellitus, vascular accidents, renal failure and gastroenteritis are the most common causes of mortality. The municipality faces a serious health risk because of the high rate of malaria in the Vhembe district. Tuberculosis was the leading cause of death among the individuals between the ages of 25–64 years. Treatment success rates remain low (62.4%) in the district.^17,18^

We retrospectively extracted data from medical records of TB patients that were initiated on treatment in all PHC facilities in the Collins Chabane municipality between April 2020 and March 2021. The data were collected from the patient records if the recorded age indicated that the patient was 17 years or older. Patient records were excluded if they had missing information that was critical for the analysis, such as the treatment outcome.

### Sample size and sampling technique

We used the Raosoft sample size calculator (Raosoft, Inc.), available online. The 1015 TB patients who started anti-TB treatment between April 2020 and March 2021 were considered the population size. We then selected a 95% level of confidence, which corresponds to a 5% margin of error, and chose a 50% response rate to obtain the minimum required sample size of 279. The final sample size was 289 after adding a 10% buffer. The percentage contribution of each of the 28 selected health facilities to the total headcount was determined and this was used to calculate the number of patient records to be sampled from each of the health facilities proportional to the required sample size. The 28 health facilities were chosen out of the 33 PHC facilities based on headcount and geographical accessibility.

The patient files were selected using the systematic random sampling strategy. The files were first ordered alphabetically by the name of patient before choosing every nth patient file until the required number of files for each health facility was achieved.

### Data entry and analysis

We used the Tier.net register to download the list of patients who met the inclusion criteria. We collected socio-demographic and clinical data using a researcher developed data collection tool, which was developed in alignment with the data elements that are captured in the TB treatment registers and the objectives of the study. The data were collected between April 2020 and March 2021.

The Excel data file was imported into the Epi Info 7.0 statistical software for data analyses. We used descriptive statistics to report the results on univariate analysis of socio-demographic and clinical data. Numeric data were presented as averages and standard deviations (s.d.). Other numeric data such as the age of patients, were further recoded into categories and reported as proportions and percentages, along with the other categorical data. These were displayed in frequency tables and graphs. For the investigation of factors associated with TB treatment success, we conducted binary and multivariate analyses, using the modified Poisson regression model. The outcome variable was dichotomised as treatment success ‘yes’ and ‘no’ categories. Treatment success was defined based on whether the patients were cured or completed the treatment, while those who defaulted the treatment, plus those who died in the course of TB treatment were categorised as having unsuccessful treatment outcome. Retreatment cases and those who died during the course of treatment were not included in the regression analysis of factors associated with treatment success.

The results of binary regression analysis were reported as unadjusted prevalence ratio (UPR), and the 95% confidence intervals (CI). All variables yielding *p* values ≤ 0.25 were included in the final multivariable Poisson regression analysis. These variables include the patients’ marital status, place of residence, HIV status, TB/HIV co-infection, other comorbidities and a treatment supervisor. The results were reported as adjusted prevalence ratio (APR). All variables yielding *p*-values < 0.05 were considered to have statistically significant association with TB treatment success.

### Validity and reliability

Prior to collection of data, the data collection tool was reviewed by two TB research experts, and the study methodologies were applied consistently with standardised processes. A pilot study was conducted using a sample of 20 patient files, and no major amendment to the data collection tool was required. The results obtained in the current study were found to be comparable to the findings of previously conducted studies. The screening of the patients’ medical files was done beforehand, and those with missing information were excluded for the record review.

### Ethical considerations

Ethical approval to conduct this study was obtained from the Sefako Makgatho University Research Ethics Committee (No. SMURE/H/68/2022: PG). Approval to conduct the study at the health facilities was granted by the Limpopo Department of Health district office and the provincial research committee of Limpopo (reference number: LP_2022-07-005). Because the study was based on retrospective data, no patients were seen and because only data from medical files were utilised, there was no disruption in the provision of services. Confidentiality and anonymity were ensured by anonymising the questionnaire and not collecting patient names.

## Results

### Socio-demographic and clinical characteristics of the tuberculosis patients

This study had a total of 289 TB patients. More than half (*n* = 183, 63.3%) were male. The mean age of the patients was 41.7 years (s.d. = 14.5). The youngest and oldest patients were 17 years and 79 years old, respectively, with 27.3% (*n* = 79) who were aged between 39 years and 49 years. More than half (66.1%, *n* = 191) were single, with 90.7% (*n* = 262) who resided in rural areas, and 90.0% (*n* = 260) who travelled ≤ 5 km to the health facility. Clinical data indicates that 97.6% (*n* = 282) of the patients were new on TB treatment. The remaining 0.4% (*n* = 7) TB cases were retreatment after failure, 1.4% (*n* = 4) and 0.4% (*n* = 1) each of relapse and retreatment after default cases and treatment after default. Of the 289 TB patients, 260 (90.0%) had pulmonary TB (PTB) and 29 (10%) had extrapulmonary TB (EPTB). Eighty-eight (30.5%) patients had been transferred from elsewhere during their course of treatment. Majority of the patients (92.7%, *n* = 286) weighed > 40 kg at the intensive start of treatment. About 40.1% (*n* = 116) had a positive HIV status, all of which were on antiretroviral therapy (ART) but with only half that had been initiated on co-trimoxazole preventive therapy (CPT) (50.0%, *n* = 58). Only 15.0% (*n* = 44) of the 289 patients had a positive smear result at 2 months, with 40.1% (*n* = 116) who had treatment supervisors and only 4.1% (*n* = 12) who had comorbidities (Table 1).

**TABLE 1:** Socio-demographic and clinical characteristics of tuberculosis patients (*N* = 289).

Socio-demographic characteristics	Frequency (*n*)	Percentage	Mean	s.d.
**Gender**				
Female	106	36.7	-	-
Male	183	63.3	-	-
**Age (years)**	-	-	41.7	14.5
17–27	60	20.8	-	-
28–38	60	20.8	-	-
39–49	79	27.3	-	-
50–60	52	17.9	-	-
> 60	38	13.2	-	-
**Marital status**				
Married	98	33.9	-	-
Single	191	66.1	-	-
**Residence**				
Rural	262	90.7	-	-
Urban	27	9.3	-	-
**Distance from the treatment centre**				
≤ 5 km	260	90.0	-	-
> 5 km	29	10.0	-	-
**Patient category**				
New	282	97.6	-	-
Relapse	1	0.4	-	-
Retreatment after default	1	0.4	-	-
Retreatment after failure	4	1.4	-	-
Treatment after failure	1	0.4	-	-
**Type of TB**				
Pulmonary TB	260	90.0	-	-
Extrapulmonary TB	29	10.0	-	-
**Transferred from elsewhere during treatment**				
Yes	88	30.5	-	-
No	201	69.5	-	-
**Body weight < 40 kg at intensive start of treatment**				
Yes	21	7.3	-	-
No	268	92.7	-	-
**HIV status**				
Positive	116	40.1	-	-
Negative	173	59.9	-	-
**TB/HIV co-infection on ART (*n* = 116)**				
Yes	116	100.0	-	-
No	0	0.0	-	-
**CPT initiation (*n* = 116)**				
Yes	58	50.0	-	-
No	58	50.0	-	-
**Baseline smear results**				
Positive	193	66.8	-	-
Negative	96	33.2	-	-
**Smear results at 2nd month**				
Positive	44	15.2	-	-
Negative	202	69.9	-	-
Not done	43	14.9	-	-
**Treatment supervisor**				
Yes	116	40.1	-	-
No	173	59.9	-	-
**Other comorbidities**				
Yes	12	4.1	-	-
No	277	95.9	-	-

TB, tuberculosis; s.d., standard deviation; HIV, human immunodeficiency viruses; ART, antiretroviral therapy; CPT, co-trimoxazole preventive therapy.

### Tuberculosis treatment outcomes stratified by patient category

Table 2 shows that 37.0% (*n* = 105) of the 282 TB patients that were newly initiated on TB treatment were cured, 29.0% (*n* = 81) defaulted treatment, 22.3% (*n* = 63) completed TB treatment, and 11.7% (*n* = 33) died in the course of treatment. Of the remaining seven TB patients who were not newly initiated on TB treatment, one was a relapsed case who managed to complete the treatment, and another one was retreated after relapsing and managed to get cured. Of the five patients who were retreated after failure, at least three were cured, with one who managed to complete treatment, and another one who died. Overall, successful treatment outcomes were observed among 60.2% (*n* = 174) of the 289 TB patients of which 37.7% (*n* = 109) were cured and 22.5% (*n* = 65) completed the treatment. Of the remaining 39.8% (*n* = 115) patients with unsuccessful treatment outcomes, 11.8% (*n* = 34) were patients that died during the course of treatment and 28.0% (*n* = 81) were those who defaulted treatment.

**TABLE 2:** Tuberculosis treatment outcome stratified by patient category (*N* = 289).

Tuberculosis treatment outcome	New cases (*n* = 282)		Relapse cases (*n* = 1)		Retreatment after default cases (*n* = 1)		Retreatment after failure cases (*n* = 4)		Total
	*n*	%	*n*	%	*n*	%	*n*	%	
Cured	105	37.0	0	0.0	1	100.0	3	50.0	109
Treatment completed	63	22.3	1	100.0	0	0.0	1	25.0	65
Died	33	11.7	0	0.0	0	0.0	1	25.0	34
Treatment defaulted	81	29.0	0	0.0	0	0.0	0	0.0	81

**Total**	**282**	**100.0**	**1**	**100.0**	**1**	**100.0**	**5**	**100.0**	**289**

### Factors associated with treatment success

As shown in Table 3, there were statistically significant associations between marital status, including the distance travelled to a health facility, and treatment supervision with successful treatment outcomes. The rates of successful treatment were higher among patients that were: male compared to the females (65.5%, *n* = 114), *p* = 0.44; single compared to those married (62.6%, *n* = 109), *p* = 0.02; residing in rural compared to urban areas (88.5%, *n* = 154), *p* = 0.32; travelled distances ≤ 5 km compared to > 5 km (96.5%, *n* = 168), *p* < 0.01; initiated treatment in the same facility compared to those who were transferred from elsewhere (73.6%, *n* = 128), *p* = 0.74; having body weight > 40 kg compared to patients who had body weight < 40 kg (94.8%, *n* = 165), *p* = 0.48; having PTB compared to those who had EPTB, (91.9%, *n* = 160), *p* = 0.17; HIV-negative compared to those whose HIV status was positive (63.8%, *n* = 111), *p* = 0.15; having other comorbidities compared to patients who had comorbidities (94.8%, *n* = 165), *p* = 0.13; and those who had supervised treatment compared to patients who did not have a treatment supervisor (60.3%, *n* = 105), *p* < 0.01.

**TABLE 3:** Bivariate analysis of socio-demographic characteristics and successful treatment outcomes among the tuberculosis patients (*N* = 255).

Characteristic	Total		Treatment success				*p*
			Yes (*n* = 174)		No (*n* = 81)		
	*n*	%	*n*	%	*n*	%	
**Gender**	-	-	-	-	-	-	0.44
Female	92	36.0	60	34.5	32	39.5	-
Male	163	64.0	114	65.5	49	60.5	-
**Age group (years)**	-	-	-	-	-	-	0.28
17–27	54	21.0	38	70.4	16	29.7	-
28–38	55	22.0	32	18.4	23	28.4	-
39–49	72	28.0	48	27.6	24	29.6	-
50–59	38	15.0	30	17.2	8	9.9	-
> 60	36	14.0	26	14.9	10	12.4	-
**Marital status**	-	-	-	-	-	-	0.02*
Married	83	33.0	65	37.4	18	22.2	-
Single	172	67.0	109	62.6	63	77.8	-
**Residence**	-	-	-	-	-	-	0.32
Rural	229	90.0	154	88.5	75	92.6	-
Urban	26	10.0	20	11.5	6	7.4	-
**Distance from the treatment centre**	-	-	-	-	-	-	< 0.01*
≤ 5 km	228	89.0	168	96.5	60	74.0	-
> 5 km	27	11.0	6	3.5	21	25.9	-
**Transferred from elsewhere during treatment**	-	-	-	-	-	-	0.74
Yes	69	27.0	46	26.4	23	28.4	-
No	186	73.0	128	73.6	58	71.6	-
**Body weight < 40 kg at intensive start of treatment**	-	-	-	-	-	-	0.48
Yes	15	6.0	9	5.2	6	7.4	-
No	240	94.0	165	94.8	75	92.3	-
**Type of TB**	-	-	-	-	-	-	0.17
Pulmonary TB	230	90.2	160	92.0	70	84.4	-
Extrapulmonary TB	25	9.8	14	8.0	11	13.6	-
**HIV status**	-	-	-	-	-	-	0.15
Positive	100	39.2	63	36.2	37	45.7	-
Negative	155	60.8	111	63.8	44	54.3	-
**Other comorbidities**	-	-	-	-	-	-	0.13
Yes	10	3.9	9	5.2	1	1.2	-
No	245	96.1	165	94.8	80	98.8	-
**Treatment supervisor**	-	-	-	-	-	-	< 0.01*
Yes	112	43.9	105	60.3	7	8.6	-
No	143	56.1	69	39.7	74	91.4	-

TB, tuberculosis; HIV, human immunodeficiency viruses.

* , Statistically significant.

Results in Table 4 show association of successful TB treatment outcomes with different predictors from modified Poisson regression model. The results from the multivariable regression analysis indicate that higher likelihood of treatment success was among patients that were single, including those who had other comorbidities and a treatment supervisor. Patients who were single had 15.0% higher likelihood of successful treatment outcomes than those who were married (APR = 1.149, 95% CI: 1.026–1.286), statistically significant *p* = 0.016. The likelihood of successful treatment outcomes was 27.0% higher among patients who had supervised treatment compared to those who did not have a treatment supervisor (APR = 1.274, 95% CI: 1.111–1.462), statistically significant *p* = 0.001. Patients who had other comorbidities also had higher likelihood of 87.0% successful treatment outcomes compared to those who had no comorbid condition (APR = 1.330, 95% CI: 1.038–1.705), not statistically significant *p* = 0.108.

**TABLE 4:** Factors associated with treatment success (*n* = 250).

Variable	Univariate Poisson regression			Mean	s.d.	Multivariate Poisson regression		
	UPR	95% CI	*p*			APR	95% CI	*p*
**Age (years)**	-	-	0.388	1.002	0.996–1.008	-	-	-
**Gender**	1.000	-	0.489	-	-	-	-	-
Male	1.067	0.887–1.282	-	-	-	-	-	-
Female	1.000	-	-	-	-	-	-	-
**Marital status**	-	-	0.014*	-	-	-	-	0.016*
Single	1.231	1.043–1.452	-	-	-	1.149	1.026–1.286	-
Married	1.000	-	-	-	-	1.000	-	-
**Residence**	-	-	0.217*	-	-	-	-	0.248
Urban	1.156	0.918–1.456	-	-	-	0.903	0.761–1.072	-
Rural	1.000	-	1.000	-	-	1.000	-	-
**Distance from the treatment centre**	-	-	0.001*	-	-	-	-	0.005*
≤ 5 km	0.304	0.149–0.619	-	-	-	0.435	0.244–0.776	-
> 5 km	1.000	-	-	-	-	1.000	-	-
**Body weight < 40 kg at start of intensive phase**	-	-	0.558	-	-	-	-	-
Yes	0.881	0.577–1.345	-	-	-	-	-	-
No	1.000	-	-	-	-	-	-	-
**HIV status**	-	-	0.161*	-	-	-	-	0.365
Positive	0.876	0.728–1.054	-	-	-	0.927	0.788–1.091	-
Negative	1.000	-	-	-	-	1.000	-	-
**Other comorbidities**	-	-	0.024*	-	-	-	-	0.108
Yes	1.330	1.038–1.705	-	-	-	1.173	0.965–1.425	-
No	1.000	-	-	-	-	1.000	-	-
**Transferred from elsewhere during treatment**	-	-	0.699	-	-	-	-	-
Yes	0.097	0.787–1.173	-	-	-	-	-	-
No	1.000	-	-	-	-	-	-	-
**Treatment supervisor**	-	-	0.000*	-	-	-	-	0.001*
Yes	1.952	1.632–2.225	-	-	-	1.274	1.111–1.462	-
No	1.000	-	-	-	-	1.000	-	-

UPR, unadjusted prevalence ratio; APR, adjusted prevalence ratio; s.d., standard deviation; CI, confidence interval.

* , Statistically significant.

Lower likelihood of treatment success was observed among patients who travelled distances ≤ 5 km to a health facility with 57.0% reduced likelihood of successful treatment outcomes compared to those who travelled distances > 5 km to a health facility (APR = 0.435, 95% CI: 0.244–0.776), statistically significant *p* = 0.005. Patients who resided in urban areas had 10.0% reduced likelihood compared to rural areas (APR = 0.903, 95% CI: 0.761–1.072), not statistically significant *p* = 0.248. Those who had a positive HIV status had 89.0% reduced likelihood compared to their HIV-negative counterparts (APR = 10.927, 95% CI: 0.788–1.091), not statistically significant *p* = 0.365.

## Discussion

Consistent with the findings from several studies, the current study found that more than half of TB patients were male.^7,19,20,21^ The study determined the proportion of patients that were successfully treated among the TB patients and found a TB cure rate of 37.7% and TSR of 60.2%. These figures are well below the targets of 85.0% cure rate and a TSR of 90.0% thresholds recommended by the WHO. Tuberculosis cure rate is the number of new smear-positive patients who are smear-negative in the last month of treatment and on at least one other occasion at least 30 days prior. Treatment success rate is the total number of new smear-positive patients who were cured and those who finished treatment but did not meet the criteria for cure or failure, divided by the number of new smear-positive PTB patients registered. Others have also reported lower rates of TB cure of 11.6% and 34.6%^22,23^ and in TSR between 57.3% and 66.8%.^10,23,24^ On the contrary, others have reported TSRs as high as 93.8%.^22^ This study found that 12.0% of patients died during TB treatment. Similarly, others have reported 12.9% and 17.0% of TB patients that died while on TB medication.^25,26,27^ Higher treatment success was observed among TB patients who were younger than 50 years of age (68.9%), those who were married (73.3%), patients who lived in rural areas (91.8%), and those who travelled distances ≤ 5 km to the health facility (83.6%).

The study further determined factors associated with TB treatment success and found a statistically significant likelihood of successful TB outcomes to be higher among single patients. This is contrary to the findings from other studies which reported good treatment outcomes among TB patients who were married compared to those who were single.^28,29^ The likelihood of successful treatment outcomes was also higher among patients with a treatment supervisor than those without treatment support. Similarly, others have reported increased TB treatment success among patients who had treatment support from community volunteers or family members compared to those without treatment support.^30,31^ Treatment supervisors play a vital role in ensuring and motivating patients to complete their treatment. The supervisor should visit patients commencing TB treatment at their homes, assess and refer other suspects and contacts to the clinic for TB screening and testing, identify problems in the household that might affect adherence and report those to the clinic.

The observed reduced likelihood of successful treatment outcomes among patients who travelled ≤ 5 km to a health facility is contrary to findings from several studies that reported a link between longer distances from a health facility and poor TB treatment outcomes. Among these is one study which reported higher odds of poor treatment outcomes among patients who travelled > 10 km to a health facility and another that reported increased risk of death among TB patients who travelled for > 2 km to a health facility.^16,32^

### Study limitations

This study was conducted in one municipality, making it impossible to generalise the findings to the entire population. The study used the retrospective record review method of data collection. There were variables that could not be collected, such as educational level, income, treatment adherence and the length of time before TB was diagnosed, which could have had an influence on the TB cure rate.

### Recommendations and implications

The TB cure rate that was observed in this study is far below the WHO target of 85%. This needs to be addressed as a national priority because it contributes to the national target. As was discovered in this research, TB patients who default on medication continue to pose a serious challenge to the efforts in TB control. Because these individuals are more likely to develop and spread drug-resistant TB strains, this issue needs to be addressed. To achieve a cure rate and avoid the establishment of TB drug resistance, health education about TB and the significance of having a treatment supporter and taking treatment on a regular basis is critical. This will also help to minimise the spread of TB in the community. Nurses at the PHC facility should provide ongoing counselling to all TB patients. Access to health care should be improved. This involves offering 24-h service at facilities and operating on weekends.

## Conclusion

The TB cure rate of 38% that was found in the study is low. The death of patients while on TB treatment is one of the biggest health concerns in this study, and this has been linked to late treatment initiation and possibly because of late presentation to a health care facility. The factors contributing to death of TB patients while on TB treatment needs to be investigated through further research. Addressing this problem in South Africa, a country which is one of the 30 countries contributing the most to the global TB burden, will not only improve the low rates in TB cure and treatment success, but this will also see South Africa play a pivotal role in advancing the global efforts to have a world free of TB, zero deaths, disease and suffering because of TB. Heightened advocacy of supervised treatment can help reduce treatment defaulting among the TB patients.
